# Repeated Application and Removal of Polyisocyanopeptide Hydrogel Wound Dressings in a Splinted Full-Thickness Wound Model

**DOI:** 10.3390/ijms24065127

**Published:** 2023-03-07

**Authors:** Roel C. Op ‘t Veld, Lieke Joosten, Peter Laverman, Ewald M. Bronkhorst, John A. Jansen, X. Frank Walboomers, Frank A. D. T. G. Wagener

**Affiliations:** 1 Department of Dentistry—Biomaterials, Research Institute for Medical Innovation, Radboud University Medical Centre, 6525 EX Nijmegen, The Netherlands; 2 Department of Dentistry—Orthodontics and Craniofacial Biology, Research Institute for Medical Innovation, Radboud University Medical Centre, Philips van Leydenlaan 25, 6525 EX Nijmegen, The Netherlands; 3 Department of Medical Imaging, Nuclear Medicine, Radboud University Medical Center, 6525 GA Nijmegen, The Netherlands; 4 Department of Dentistry, Research Institute for Medical Innovation, Radboud University Medical Centre, 6525 EX Nijmegen, The Netherlands

**Keywords:** polyisocyanopeptide, hydrogel wound dressing

## Abstract

Polyisocyanopeptide (PIC) hydrogels are proposed as promising wound dressings. These gels are thermo-sensitive, allow application as a cold liquid, and rely on gelation through body heat. It is supposed that the gel can be easily removed by reversing the gelation and washing it away with a cold irrigation solution. The impact on wound healing of the regular application and removal of PIC dressings is compared to a single application of PIC and the clinically used Tegaderm™ in murine splinted full-thickness wounds for up to 14 days. SPECT/CT analysis of ^111^In-labelled PIC gels showed that, on average, 58% of the PIC gel could be washed out of the wounds with the employed method, which is, however, heavily influenced by personal technique. Evaluation with photography and (immuno-)histology showed that wounds in which PIC dressings were regularly removed and replaced were smaller at 14 days post-injury but performed on par with the control treatment. Moreover, the encapsulation of PIC in wound tissue was less severe and occurred less often when PIC was regularly refreshed. In addition, no morphological damage related to the removal procedure was observed. Thus, PIC gels are atraumatic and perform similarly to currently employed wound dressing materials, offering possible future benefits for both clinicians and patients.

## 1. Introduction

Skin wounds have varying severity levels and originate from trauma or surgery or develop slowly over time, as with pressure ulcers. The subsequent healing of skin wounds can be suspended, delayed, or diminished for various reasons. For example, re-injury can occur, after which healed structures are lost, and the healing progress is reset. Alternatively, a chronic wound remains in the inflammatory phase, preventing the resolution of inflammation and (myo)fibroblasts from closing and restoring the skin. In the case of burn injuries, underlying structures, such as nerves and sweat glands, can be so profoundly damaged that their function is completely lost. At the same time, the patient simultaneously ends up with permanent scarring. The use of advanced wound dressings is recommended for the treatment of such complex wounds [1,2]. Besides acting as a cover for the wounded area, wound dressings can include valuable functionalities, such as protection against bacteria, absorption of wound exudate, or keeping the wound hydrated [3,4,5,6]. This may help properly heal the wound and prevent scarring or other complications. However, the production costs and the amount of money and time healthcare personnel require to apply and refresh wound dressings are tremendous. Recent studies on the economic burden on the United Kingdom’s National Health Service (NHS) showed that the cost of treating a wound in the clinic ranges from GBP 698 to GBP 3998. These costs increase by 35% on average when the wound remains unhealed [7,8]. Changing wound dressings are often painful, especially for burn victims. Thus, it should be evident that better wound dressing products are desired to improve wound treatment and help healthcare providers and patients by making it more convenient to apply and remove painlessly.

Recently, hydrogel wound dressings based on polyisocyanopeptide (PIC) polymers were reported as a potential product for treating skin injuries [9,10]. The benefit of this product comes from its reversible thermosensitive gelation behaviour and chemically modifiable polyethylene glycol (PEG) side chains [11,12,13,14,15,16]. The envisioned clinical application method involves a PIC solution that is cooled into the liquid state (<15 °C, although this is tuneable [11]) and then poured or sprayed onto the wound. The product will flow into every corner of the wound and subsequently undergo in situ gelation triggered by body heat, allowing the PIC gel to form a protective barrier. While present, the gel supposedly prevents bacterial entry, hydrates the wound, and provides additional (bio-)functionalities, such as drug delivery [9,17]. Other material benefits of the PIC hydrogel include an extracellular matrix-mimicking strain stiffening response, adjustable stiffness, rheology and pore size, and the possibility of chemical functionalization [9,10,15,18].

In the clinic, burn injuries are typically washed with a shower head or similar irrigation techniques daily. At this moment, PIC gel may likely be conveniently and painlessly removed, as its largely water-based origin allows it to be flushed out easily. Furthermore, if the irrigation fluid’s temperature is below the gelation point, the gel may reverse back to liquid, further improving the efficiency of this step. Pain-free removal of a dressing is desirable, as the debridement and dressing changes performed in burn wound care are extremely painful to the patient but, unfortunately, necessary to prevent infection [19]. Moreover, hydrogels such as PIC are lowly adherent and more likely to be atraumatic [20]. Atraumatic dressings do not cause additional damage to the wound upon removal, and this lack of disturbance to the wound repair process is overall beneficial to the outcome [21]. It was therefore postulated that the convenient application and removal of PIC dressings might facilitate wound repair by eliciting less disruption.

In this current study, the feasibility of the aforementioned application-and-removal approach is simulated in a splinted wound model in mice. Previously, it was determined that PIC gel may provide a microbial barrier function [9] but may eventually become encased below the skin surface in the more deeply positioned tissues [10]. Currently, it is unknown whether irrigation of the wound will effectively wash out PIC gel and if this leads to undesired adverse effects such as morphological damage or delayed healing. Furthermore, it remains to be answered how treatment with PIC gel impacts the healing of wounds for a period longer than seven days. Therefore, hairless immuno-competent mice were subjected to a splinted full-thickness skin defect, treated with PIC gel, and a secondary dressing. This model was chosen for its ease of use and controllable environment, typically resulting in undisturbed wound healing. Experimental groups included wounds receiving 1. a single dose of PIC gel after surgery and regularly subjected to a washing procedure and new doses of PIC gel, and 2. wounds treated only with a non-adhesive Tegaderm™ sheet and a bandage were used as a control group that simulated present clinical treatment routines [22,23]. The study consisted of 3 parts; first, an initial study in which the washing procedures were practised, and the wound healing status was assessed at 14, 21, and 28 days post-injury. Second, a SPECT/CT imaging study, in which the efficiency of removing ^111^In-labelled PIC-DTPA gel from wounds by cold saline rinsing was quantified. Third, a main wound healing study where the impact of a single or repeated dose of PIC hydrogel on wound size was analysed at 3, 7, and 14 days post-injury. The outcome of wound repair of these treatments was assessed by photography and (immuno-)histology. It was hypothesised that PIC gel does not interfere with wound healing up to 14 days and that a regular washing schedule does not interfere significantly with wound repair and prevents the encapsulation of PIC gel in the wound repair tissue.

## 2. Results

### 2.1. Animal Welfare

In general, discomfort and pain were lower than anticipated. Although, in the initial study, it was noted that mice could develop frostbite injuries on the tail as a result of this washing procedure. In the main wound healing study, two mice died prematurely. One of these mice suffocated due to the secondary dressing being applied too tightly, which impeded breathing. This mouse could be transferred to another treatment group without consequences. The other mouse died because of undercooling during the washing procedure. It was decided to re-allocate one mouse from the day three control group to this group (14 days refreshed PIC group). As a result, all groups in the main wound healing study were *n* = 6 except for the day three control group (*n* = 5). Furthermore, more attention was paid after this incident to maintaining the mice’s body temperature during the washing procedure to prevent future loss.

### 2.2. Identification of the Late-Stage Healing Time Point (Initial Study)

The initial study was done to obtain experience and insights in correctly applying the primary and secondary dressings and practising the washing procedure. In addition, histological analysis of the skin injury was performed to assess the most appropriate time point of the remodelling phase for further experiments. It was found that identification of the wound area on days 21 and 28 was challenging. Most wounds were healed to the point where comparing inter-treatment effects became too difficult. In contrast, wounds of day 14 showed thick wound repair tissue with remodelling evidently in effect. It was therefore decided to follow mice in the main wound healing study until day 14.

Moreover, HE-stained sections displayed accumulations of inflammatory cell types. A follow-up with a Von Kossa staining revealed that calcified deposits were present within the wound repair tissue. These deposits were first observed on day 14 in low quantities and only in some specimens. More deposits were found in sections of days 21 and 28. Moreover, giant cells were observed in low numbers, mostly in samples of days 21 and 28. Histological images showing the calcifications are provided in Supplementary Figure S1.

### 2.3. Efficiency and Effect of Washing Procedures (Imaging Study)

#### 2.3.1. Determining the Washing Efficiency

**Figure 1:**
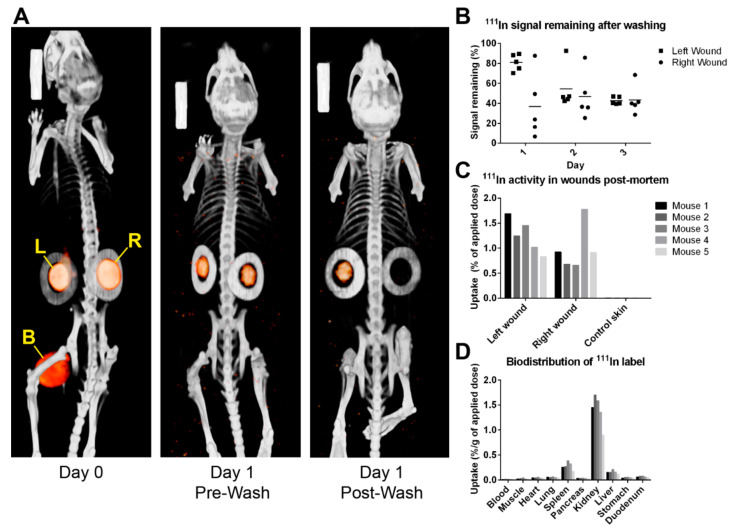
[^111^In]In-DTPA-PIC gels applied to splinted wounds were tracked by SPECT/CT imaging before and after washing. (**A**) SPECT/CT scans show the orange signal (SPECT) originating from PIC gel within the circular-shaped splints (greyscale CT signal) of the left wound (L) and right wound (R). After washing (on day 1), the SPECT signals in the wounds decrease. In the left wound, the signal becomes visually lower, while the signal from the right wound is so low that it is no longer visible after image processing. The large signal source between the hind legs on the day 0 scan originates from ^111^In-bound EDTA in the bladder (yellow B). (**B**) This dot plot indicates the amount of ^111^In signal remaining in the wound areas after washing via different methods (see text), as quantified from SPECT/CT scans. Horizontal bars represent mean signal remaining. (**C**,**D**) Post-mortem activity measurements still show high activity in the wounds, the kidneys, and a small amount in the spleen and liver. Activity in wounds was expressed as a percentage of the applied dose instead of the applied dose per gram of weight because samples were too light to weigh accurately.

The application of PIC gel to the wound was easily achieved, and the signal originating from the PIC gel was imageable by SPECT/CT immediately after application and the day after that, similar to our previous study [10]. The amount of signal remaining in the wounds was expressed as a percentage of the signal before washing. This value is referred to as the ‘remaining’ signal. The results are summarised in Figure 1. On the first day, it was discovered that the washing strategy using a 50 mL syringe was not sufficiently effective. It was observed that PIC gel filled the full-thickness pocket, and most saline bounced off the PIC gel during rinsing.

Furthermore, a dichotomy between the left and right wounds was found. The left wound had a remaining signal of 80.9 ± 8.3%, while the right wound had 36.8 ± 32.5%, indicating the importance of the animal’s and researcher’s positioning during washing. Therefore, on the second day, mouse 1 was washed using a 10 mL syringe (applied volume was kept consistent at 25 mL). It was immediately observed that this was ineffective, and the remaining signal was considerably high at 92.5% (left) and 85.7% (right). The methodology was changed by switching back to the 50 mL syringe equipped with a cannula that improved precision and pressure. The remaining signal improved to 45.0 ± 3.8% and 37.2 ± 10.4%, respectively. On the third and final day, the remaining signal was 42.6 ± 3.8% (left) and 43.4 ± 14.8% (right), respectively. The washing method deemed feasible enough for the main wound healing study, namely using a 50 mL pipette with cannula, averaged a remaining signal of 42.2 ± 9.1%. The discrepancy between left- and right-positioned wounds also disappeared by adjusting the washing method. Biodistribution studies showed high activity in the excised wounds but not on healthy dorsal skin. This elevated activity was also evident in the kidneys, spleen, and liver.

#### 2.3.2. Histological Investigation of Washing Procedures

**Figure 2:**
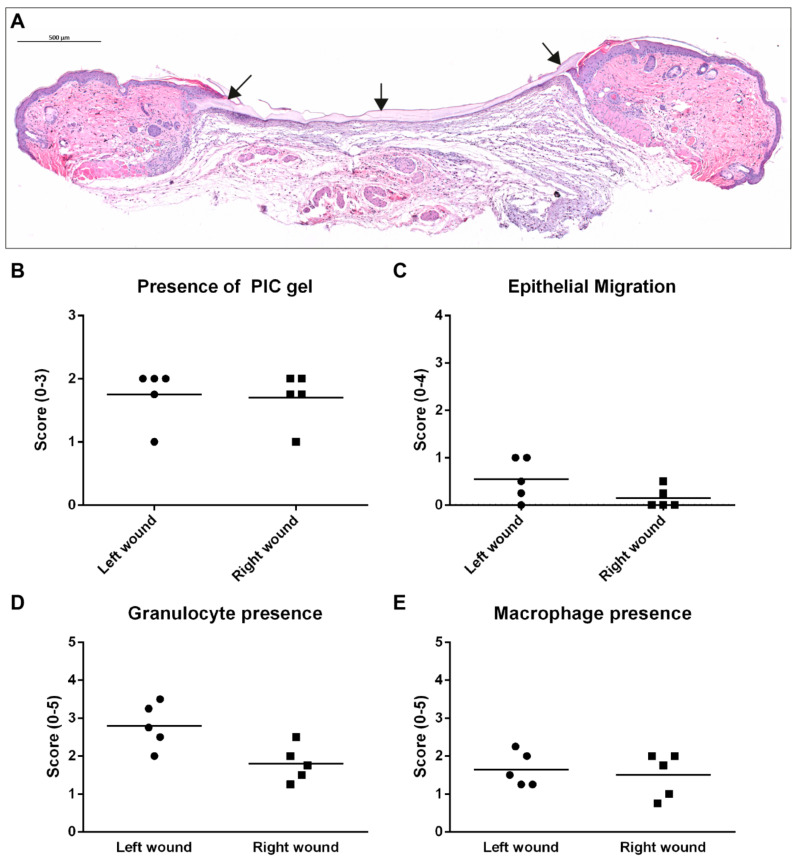
Results of histological analysis in the imaging study, taken at three days post-injury. A distinction was made between left and right wounds. (**A**) This HE-stained section is positioned closer to the edge of a wound and shows a clear layer of PIC gel (indicated by black arrows) on top of the wound even after the washing procedure. Scale bar indicates 500 µm. (**B**) The presence of PIC gel was scored in all wounds and was mostly awarded with a score 2 (large volumes of PIC gel positioned topically). (**C**) The migration of epithelium was mostly not initiated yet on day 3. (**D**) More granulocytes were present in left-wounds compared to right-wounds on average. Total granulocyte scores were also higher compared to the main wound healing study. (**E**) The presence of macrophages was similar in left and right wounds.

HE-stained sections from three days post-wounding were analysed for the presence of PIC gel and morphological damage that could be related to the treatment or washing procedure. The results are presented in Figure 2A–C. Every wound showed the presence of PIC gel. In two wounds, the volume of PIC was considered low and dispersed scarcely, while in the remaining wounds, PIC could be observed mainly in the wound edges as a layer lying on top of the wound. As can be expected from wounds three days post-wounding, epithelial migration was mostly not initiated. No distinct morphological changes or damage were observed related to the PIC gel. Next, immuno-histological stainings were scored for the presence of granulocytes and macrophages (Figure 2D,E). Granulocytes were present in significantly higher numbers in left than right wounds (*p* = 0.044), while the number of macrophages was comparable (*p* = 0.646). Myofibroblasts were not yet present on day 3.

### 2.4. Effect of PIC Treatment on Wound Healing (Main Wound Healing Study)

#### 2.4.1. Wound Closure Is Not Affected by PIC Treatment

**Figure 3:**
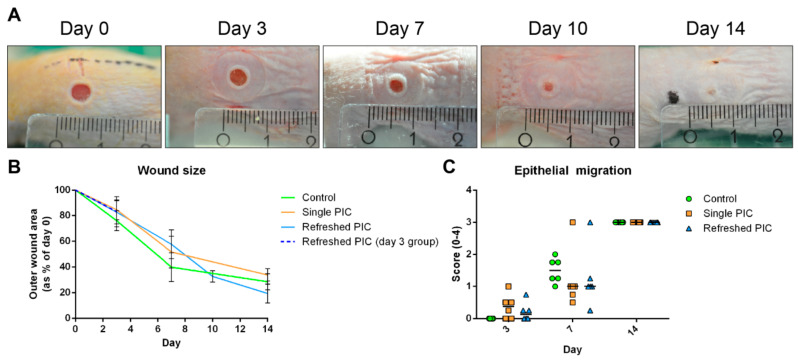
Wound size and the migration of epithelium. (**A**) This photo series shows the closure of the left wound on a mouse from the day 14 refreshed PIC group (smallest ruler increments in millimeters). The wound closes, and epithelium migrates between days 7 and 14. (**B**) Quantifying the outer wound sizes using ImageJ on photographs (error bars represent standard deviations). The day 3 refreshed PIC group is depicted separately, as the protocol deviated slightly from the other time points. PIC-treated wounds were significantly larger on day 7, whereas refreshed PIC wounds were significantly smaller on day 14. (**C**) Migration of epithelium was scored on HE-stained sections and typically initiated between days 3 and 7 (horizontal lines represent the medians). By day 14, all wounds were closed, and keratinization had taken place. No hypertrophic wounds were found.

The wound closure is summarised in Figure 3. The rate of wound closure was comparable for all groups at three days post-injury (Figure 3B). On day 7, wounds from single PIC and refreshed PIC groups were significantly larger than the control (*p* = 0.002 and *p* < 0.001, respectively). However, on day 14, wounds in the single PIC group were of comparable size as the controls. By contrast, wounds in the refreshed PIC group were significantly smaller than the other treatments (*p* = 0.004 and *p* < 0.001 for control and single PIC, respectively). Next, the migration of epithelium, and possible hypertrophic developments, were scored in HE-stained sections (Figure 3C). For most wounds, epithelial migration was initiated by day 7 and completed on day 14 (score 1). This was in accordance with photographic evidence, where the epithelialised area can be distinguished from the non-epithelialised area based on the colour. No significant differences were found between treatment groups’ rates of epithelial migration at any time point.

#### 2.4.2. Gel Presence and Wound Morphology

**Figure 4:**
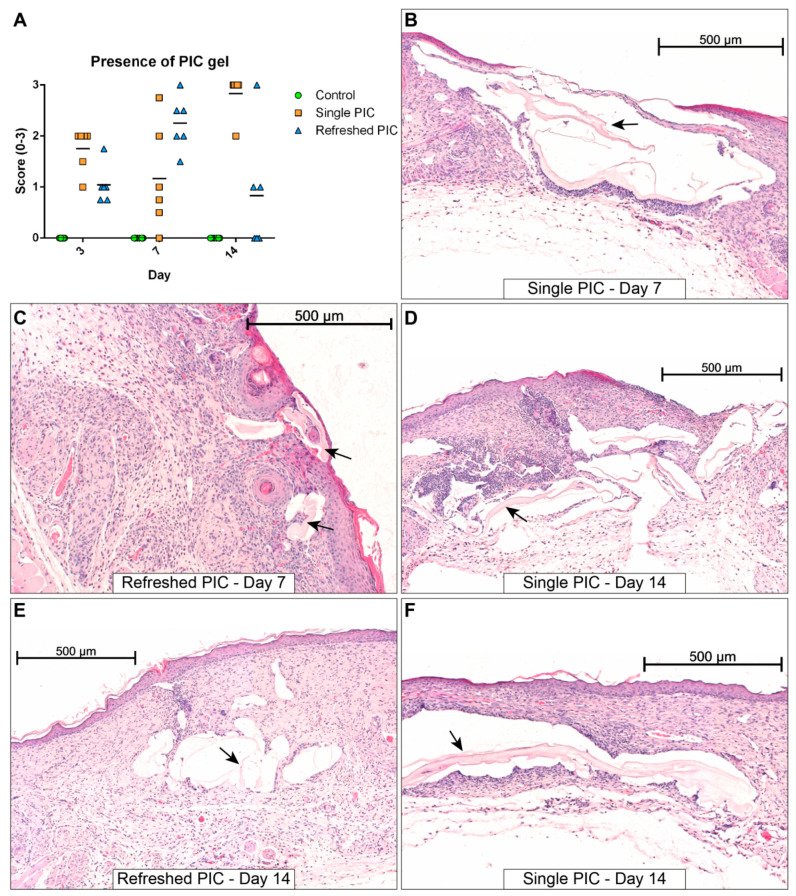
Overview of HE-stained wound sections with traces of PIC gel (indicated by black arrows). (**A**) The presence of PIC gel in samples was scored on a scale of 0 to 3 (horizontal lines indicate the medians). The gel tends to be larger in volume in the single PIC group (**B**) compared to the refreshed group (**C**). The structural damage related to PIC gel tends to be smaller for the refreshed PIC group (**E**) compared to the single PIC group (**D**,**F**). Scale bars indicate 500 µm.

HE-stained sections were analysed for the presence of PIC gel or remnants thereof. The scoring of gel remnants and several interesting histological sections depicting PIC gels are shown in Figure 4. On day 3, when wounds were still large and open, the PIC gel was observed in both the single and refreshed PIC groups. The volumes in the single PIC group appeared visually bigger, but there was no statistically significant higher score in the presence of gel (*p* = 0.080). On day 7, this group’s PIC volumes were still visually larger. Still, the refreshed PIC group displayed a higher average score in gel presence, indicating a higher incidence of encapsulated PIC. However, this was not a statistically significant increase (*p* = 0.061) due to one outlier in the single PIC group causing a large deviation.

On the other hand, the gel tended to be positioned more topically on the wound in the refreshed PIC group. The most notable differences occurred on day 14, where it could clearly be observed that samples from the refreshed PIC group had fewer and smaller PIC gel-containing compartments in the wound repair tissue than the single PIC group. This effect was statistically significant (*p* = 0.015). As shown in Figure 4, these PIC gel remnants interfered with proper formation/remodelling of the wound repair tissue, as indicated by the open gel-filled compartments interrupting the continuity of the wound repair tissue. No other morphological changes were observed besides those related to the PIC gel. Giant cells were only observed on day 14, but in fewer numbers than in the initial study. An overview of HE-stained sections of each time point and treatment is given in Supplementary Figure S2.

#### 2.4.3. Evaluating Inflammatory and Proliferation Phase Markers with (Immuno-)Histology

**Figure 5:**
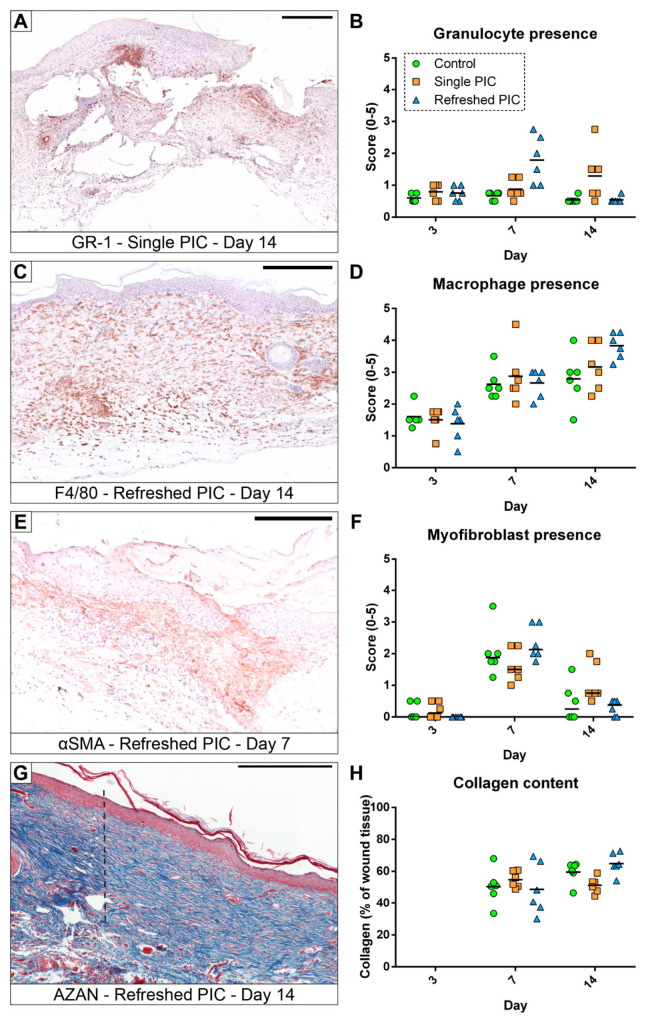
Representative histologically stained sections and the quantitative results of the associated analyses. (**A**,**B**) Granulocytes represented only a small percentage of the wound tissue but are increased in several specimens. (**C**,**D**) Macrophages were present in each phase of the wound repair, the influx was observed from nearby healthy tissue, and they were a dominant cell type, especially at later time points. (**E**,**F**) The differentiation of fibroblasts into αSMA-positive myofibroblasts occurred mainly in the proliferation phase (day 7, (**E**)). (**G**,**H**) The percentage of blue-positive collagenous tissue was quantified digitally; in the depicted section (**G**), the transition from the dark-blue highly organised collagen in healthy tissue into the lighter-blue less organised collagen in the wound repair tissue is very apparent (indicated by the dashed black line). The scale bars indicate 250 µm, and the horizontal lines represent the means in all graphs except the myofibroblast presence, where the median is indicated.

Immuno-histologically sections stained for GR-1 (granulocyte marker), F4/80 (macrophage marker), and αSMA/Actin2 (myofibroblast marker) were assessed for the presence of the specifically stained cell type (see Figure 5). All groups had low granulocyte presence on day 3, with scores between 0.5 and 1.0. A notable increase in the number of granulocytes occurred in the refreshed PIC group on day 7 compared to the control (*p* = 0.002) and single PIC group (*p* = 0.011). This effect dissipated on day 14, where the single PIC group showed significantly more granulocytes than the other groups (*p* = 0.043 to both).

Macrophage presence was higher on day 7 for all groups and stayed constant through day 14, except in the refreshed PIC group, which increased further. However, no significant differences were observed between groups, although it has to be noticed that the *p*-value for the refreshed PIC group was 0.051.

Myofibroblasts were absent on day 3, but their amount was increased on day 7 during the proliferation phase and was reduced again on day 14 during the remodelling phase. Non-parametric testing showed a significant increase in myofibroblast presence in the single PIC group compared to the refreshed PIC group on day 14 only (*p* = 0.035).

The percentage of collagen attributing to total tissue was not calculated for day 3 specimens, as the size of the full-thickness wounds were still very large, and most samples did not contain sufficient wound tissue for analysis. Due to this lack of wound tissue development, the collagen content could not be measured at that time point. On day 7, the percentage of collagen was comparable in all treatment groups (*p* = 0.655). On day 14, collagen content in the single PIC group was similar to that in the control group (*p* = 0.085) but significantly lower than in the refreshed PIC group (*p* = 0.005).

## 3. Discussion

This study explored the feasibility of PIC hydrogel as a removable wound cover. The removal of the gel from wounds by washing with a cold solution was demonstrated. The impact of the material on wound repair was assessed at three critical moments during wound healing, namely during the inflammatory phase (day 3), proliferation phase (day 7), and remodelling phase (day 14). It was found that wounds treated with PIC gels performed on par with control wounds and the overall treatment benefits from regular dressing replacements. 

Firstly, an initial study was performed to explore the wound model and washing procedures and identify the most appropriate time point representing the remodelling phase. Moreover, it was found that histological samples taken on days 21 or 28 after surgery showed such advanced healing that it was too difficult to identify where the original wound was made. Therefore, it was decided to follow up on wounds until day 14 in the main study to ensure that a reliable comparison between groups could be made. Moreover, it was discovered that PIC polymers that remained in the wound repair tissue calcified and triggered an immune response on days 14, 21, and 28. These reactions are undesirable, as heterotopic calcification can lead to additional complications [24]. Moreover, giant cells were identified in small numbers around these calcifications. So far, it remains unclear whether these giant cells arrive first and trigger the calcification or vice versa. It is postulated that this process is linked to the dissipation of PIC’s liquid component (PBS), as this response has only been observed for 14 days or later in sections where the polymer is left behind. 

Further research has to be performed to determine the long-term response to PIC gels/polymers and to discover if PIC polymers are degraded, expelled, or stay behind in the tissue. To that end, PIC’s material characteristics will play an important part because of its mimicry of natural extracellular matrix characteristics, such as stiffness and strain-stiffening, which may help prevent a negative host response [25].

Tuning gels by adding biofunctional or chemical groups have also been shown to change the gel behaviour and properties in other model systems, which may influence cell–gel interactions, drug delivery, or wound repair [18,26,27,28,29]. Here, an imaging study was performed using ^111^In-labelled PIC gel to determine the efficiency of gel removal from the wounds by cold saline washing. First, it was found that the ^111^In-DTPA PIC hydrogel could be easily detected using SPECT/CT. Based on results obtained here and in our previous study [10], it is assumed that the signal present in the wounds 24 h after application of the labelled gel originates solely from the [^111^In]In-DTPA-PIC. After this period, EDTA-bound ^111^In is expected to be cleared through the urine, and any remaining detectable signal should originate from PIC-bound ^111^In. Still, there is a possibility that some part of the signal originates from anything other than the PIC, such as radiolabelled polymer fragments or EDTA. However, this source is expected to be insignificant compared to the gel. 

In addition, we explored different ways of washing to be used for the main study. The study showed that after adjusting the washing method, on average, 40 to 45% of PIC gel remained in the wounds after washing. The original intention was to consistently remove at least 90%, which was only achieved once. It was found that at these early time points the full-thickness wounds represent a very stringent model by having a deep pocket that retains the washing saline and gel like a reservoir. The washing saline was sometimes observed bouncing off the wound, making it difficult to remove all of the PIC gel, especially from the deeper corners. Since humans do not have loosened skin like mice, full-thickness wounds will likely have less gel remaining in the skin upon washing. Partial-thickness wounds would abolish this problem altogether. The remaining gel would also be pushed out because stem cells in the partially wounded skin would keep proliferating and differentiating.

In this study, it was chosen to use cold saline in the washing procedure. It was postulated that the temperature-induced switch of PIC from a gel into a liquid would facilitate easy removal. It remains unclear if the cold drop of saline added to the wounds before washing was sufficient to induce this transition. Moreover, the temperature employed (~4 °C) in this current study can be stressful, discomforting, and even delay wound repair in animals and patients [30]. In a clinical setting, a good strategy may instead include first cooling the wound area with a “cold packing” before washing (thoroughly) with (luke)warm water. This could trigger the transition of PIC into a liquid, enabling an easier washout without being detrimental to the patient. Nevertheless, we decided to continue the cold washing in the main wound healing study, as we did not want to change the original study design. Still, a conscious effort was made to wash the wounds more efficiently by adjusting the saline stream angle and aiming into each side of the wound. 

For the main wound healing study, the rate of wound closure and general tissue morphology were first assessed using photography and HE-stained sections. Photographic analyses showed that PIC-treated wounds were larger than their control counterparts on day 7. However, on day 14, the wounds in the refreshed PIC gel group were significantly smaller than the control and single PIC gel groups. This suggests that PIC gel or the washing procedure can accelerate wound closure. Re-epithelisation occurred at comparable rates in all groups, indicating that the PIC gel or washing procedures did not impede this process. 

Furthermore, a previous study showed that PIC gel could become encased in the wound repair tissue [10]. Part of the current study was to discover if regular wound-washing steps can help prevent this phenomenon. By washing away the old PIC gel and applying the new dose topically, it was postulated that PIC gel was less likely to get trapped in the more deeply positioned tissues. Histological evaluation of the imaging study showed minimal wound repair tissue development by day 3. Additionally, although PIC was still present, it was only observed to be positioned topically and not encapsulated in the wound tissue. The main study showed that wounds in the refreshed PIC group on day 14 displayed smaller and less encapsulated PIC gel than the single PIC group. By contrast, on day 7, the presence of PIC gel scores was, on average higher in the refreshed PIC group than in the single PIC group. In addition, on day 3, it was found that wounds in the imaging study typically had more PIC gel (average score 1.7) than wounds in the main healing study (average score 1.2). Both these findings can be attributed to new doses of PIC gel that were recently applied to these wounds. This effect disappears on day 14 because the wounds have closed considerably at this point, and the newly applied doses of PIC gel are positioned more topically where they are more easily washed away. Importantly, the formation of large, encapsulated compartments of PIC gel on day 14 occurred significantly less often in the refreshed PIC group. This suggests that, even though PIC gel is sometimes observed in larger volumes in earlier time points, the washing and replacement procedure may help prevent encapsulation of the gel and possibly reduce the odds of calcified deposits in the long term.

Furthermore, the washing procedures did not induce additional morphological damage and, on average, looked less structurally interrupted by PIC gel than wounds from the single PIC group. This suggests that PIC gels are atraumatic, i.e., do not cause damage to the wound bed upon removal [20,21]. This is a major benefit, as removing most dressing materials is painful. It has been shown to damage the newly formed epithelium and peri-wound area, which increases wound size and delays healing, especially when performed repeatedly in, e.g., chronic wounds [31]. Lastly, the calcifications observed in the initial study were no longer observed in the main study. Additionally, the inflammatory responses, including giant cells, were less severe and frequent. 

Next, immunohistologically and AZAN-stained sections were analysed for relevant wound repair cell types and collagen deposition, respectively. In general, granulocyte numbers were low. At day 3, granulocytes were also considerably lower in all groups of the main study compared to the imaging study. This possibly relates to introducing de novo contaminants during the DTPA-PIC-labelling procedure triggering an immune response. However, in the main study, an increased granulocyte influx was found for the refreshed PIC group on day 7 and the single PIC group on day 14. This can likely be attributed to immune responses against the foreign material rather than immune responses involved in wound repair. However, it must be stressed that no infections were noted in any mouse and that the overall presence of granulocytes was still considered low. Moreover, it was found that the wound tissue contained a considerable number of macrophages at all time points, but no significant differences were observed between the groups. Macrophage levels at day 3 were also highly comparable between the imaging and the main study.

Furthermore, using a non-parametric test, the single PIC group displayed significantly higher myofibroblast numbers on day 14 than the refreshed PIC group. Although this effect is only minimal, it suggests a slightly delayed healing in the single PIC group. Lastly, the treatments resulted in a comparable collagen deposition throughout the study. Only on day 14, a significant reduction in collagen expression between single PIC compared to refreshed PIC was found. This may be related to the deposition of connective tissue around PIC gels, which was present in larger volumes in this group at this time point. Overall, for the inflammatory and proliferation phase markers, wounds subjected to the dressing refreshment protocol performed similarly to the control group but better than those treated with a single dose of PIC. It was attempted to validate these findings with gene expression analysis using qPCR. However, while processing the results, it was concluded that the data were unreliable, as a portion of the harvested tissue included healthy cells. This was especially so for day 3, where healthy skin composed a larger percentage of the harvested tissue, clouding the wound cells’ expression patterns.

Finally, a limitation in this current study was the murine model. The mice’s small size and loose skin make it difficult to wash and remove the PIC gel effectively from the created full-thickness skin wounds. As was observed previously, PIC gel can leak easily into the subcutaneous layers of this mouse model after establishing a full-thickness skin wound and likely remain behind [10]. Therefore, it is recommended that for follow-up studies, an animal model is used with a skin structure more similar to the human situation (e.g., the pig). Nevertheless, the murine model allowed the assessment of the initial impact of PIC hydrogel, applied either once or repeatedly, on wound healing up to two weeks. The obtained data provide reasonable evidence to confirm the merit of PIC hydrogel as a wound dressing. No apparent harmful interference with wound repair was found related to PIC gel or its removal, which opens options for future development into a clinically relevant product. In particular, PIC gels may be appropriate in hydrating (dry) burn wounds, and further molecular modification to introduce therapeutic options is encouraged. 

## 4. Materials and Methods

### 4.1. Material Preparation

Triethylene glycol-grafted PIC polymers (Radboud University, Nijmegen, the Netherlands) were synthesised and prepared as described in detail previously [9,10,15,18,32]. Synthesis of DTPA-functionalized PIC polymers (purchased from Noviocell BV, Oss, The Netherlands) and radioactive labelling with ^111^In was performed as described previously [10]. For the imaging study, one batch of ^111^In-labelled PIC-DTPA gel was prepared in metal-free 2-(N-morpholino)ethanesulfonic acid (MES, Sigma Aldrich, Saint Louis, MI, USA) buffer at a final concentration of 4 mg/mL. For the main wound healing study, one large batch of PIC hydrogel was created at a concentration of 4 mg/mL using sterile PBS (Thermo Fisher Scientific, Waltham, MA, USA), which was aliquoted and stored at −20 °C for the duration of the experiment. The synthetic PIC hydrogel is a novel synthetic and thermoresponsive (liquid below 16 °C; gel above 16 °C) hydrogel. Detailed information about the morphology, pore size, and rheology can be found in the following references [9,10,15,18].

### 4.2. Animals

A total of 65 male mice were used, and the study was approved by the Committee for Animal Experiments of the Radboud University, Nijmegen, the Netherlands (DEC 2016-0103), and the guidelines set by the European Union in directive 2016/63/EU were followed. All mice were of strain SKH-1 (hairless) and supplied by Charles River Laboratories (Sulzfeld, Germany); all experiments were performed at the Central Animal Laboratory (CDL) of the Radboud University. Fifty-four animals were used in the main healing study, five for imaging with SPECT/CT and six for initial experiments. Mice were between 6 and 7 weeks of age at the start of the experiment and were housed individually in a temperature-regulated room (25 °C) on a 12 h light–dark cycle with fed food and water ad libitum.

### 4.3. Splinted Full-Thickness Wound Model

Mice were anaesthetised using an evaporated isoflurane/air mixture. Analgesia was provided (5 mg/kg Rimadyl, Zoetis Inc., Kalamazoo, MI, USA) half an hour before surgery and 24 h later. The dorsal skin was disinfected using Betadine (Meda Pharma B.V., Amstelveen, The Netherlands). Then, two full-thickness skin wounds were punched out with a Ø 4 mm biopsy punch (Kai medical, Seki City, Japan) by stretching a piece of folded dorsal skin and pressing the punch through all skin layers as described previously [33]. Silicone ring splints (inner Ø 6 mm, outer Ø 12 mm, thickness 0.5 mm) were glued around the wound using cyanoacrylate gel (Bison, Goes, The Netherlands). While the glue was drying, 20 µL PIC gel was defrosted and applied directly to the wound in the cold liquid state using a pipette. After gelation of the gel was established, the wounds were covered in a cut-to-size sheet of Tegaderm™ (3M Medical, Diegem, Belgium) and a single wrap of Petflex^®^ no-chew bandage (Andover Healthcare Inc., Salisbury, MA, USA) covering only the mouse’s underbelly and back. For animals included for 14 days or longer in the experiment, these splints and secondary dressings were replaced (under anaesthesia) at 7 days after surgery. For the imaging study, [^111^In] In-DTPA-PIC gel was kept defrosted on ice in a lead container.

### 4.4. Washing Protocol

To wash PIC gel out of the wounds, mice were placed under anaesthesia on a heating mat warmed to 37 °C. First, a droplet of fridge-cooled (~4 °C) sterile saline solution (Braun GmbH, Kronberg, Germany) was added to each wound to attempt reversing the gelation of the gel over a period of roughly one minute. Then, 25 mL of the cold saline was used for flushing and rinsing each wound. This method was performed using a 50 mL syringe (BD Plastipak™, Franklin Lakes, NJ, USA) with (18G Monoject™, Covidien, Tullamore, Ireland) to improve precision and pressure. The imaging study also attempted the method without a cannula but with a 10 mL syringe (BD Plastipak™). Attention was paid to changing the angle and pressure so that each corner of the wound was rinsed out properly. After washing, the cold saline-soaked padding was removed, and the body temperature was recovered with a heating lamp. During this time, new splints were glued in place. Subsequently, the lamp was turned off, and a new dose of PIC gel and the secondary dressing were re-applied.

### 4.5. Study Designs

The present study involved three sub-studies involving the aforementioned splinted full-thickness wound model (see Table 1).

In the initial study, six mice received full-thickness skin wound injury and were treated with PIC gel and a secondary dressing. Three of the six mice underwent the washing protocol every third and fourth day in sequence, applying a new dose of PIC gel each time. Additionally, 2 mice (1 washed and 1 non-washed) were euthanised on days 14, 21, and 28 to determine the suitability of these time points as an indicator of wound healing conditions in the remodelling phase. Both wounds were harvested and processed for histological staining.

In the imaging study, five mice were subjected to the splinted full-thickness wound model and daily washing procedures up to three days post-injury. On day 0, the mice were operated, treated with the ^111^In-labelled PIC gel, and scanned with SPECT/CT. On the first and second day after applying PIC gel, the mice were imaged before and after washing the wounds, and a new dose of PIC was applied. Finally, on the third day after wounding, the protocol was repeated, but the animals were euthanised after the final scan and did not receive a new dose of PIC gel. The organs were harvested for biodistribution analysis, and subsequently, both wounds were processed for histological staining.

In the main wound healing study, 54 mice underwent the splinted full-thickness injury model. Mice were randomly assigned over three treatment groups and three time points using an online random number generator (www.random.org, accessed on 21 November 2017). The ‘control’ group was a sham treatment in which the wounds were created, but mice only received a secondary dressing (Tegaderm™ and bandage) and no PIC gel. In the ‘single PIC’ group, mice received a single dose of PIC gel per wound and a secondary dressing following surgery. In the ‘refreshed PIC’ group, 20 µL of PIC gel and a secondary dressing was applied after wounding. Then, the mice underwent the washing protocol on days 3, 7, 10, and 14, ensuring that a fresh dose of PIC gel was always present. From each group, 6 mice were euthanised on days 3, 7, and 14 post-injury. An exception to the protocol was in the refreshed PIC group on day 3. The wounds of these mice were washed before harvesting to distinguish their treatment from the day 3 single PIC group. This contrasts with the refreshed PIC group on days 7 and 14, where wounds were not washed before harvest. One wound was processed for histological analysis, and the other was intended for gene expression analysis.

### 4.6. Wound Size Analysis

Photographs of the wounds were taken after surgery and sacrifice and at any intermediate point when bandages were uncovered (e.g., when washing or replacing the dressing). To this end, Tegaderm™ sheets had to be removed, typically causing splints to become undone as well. Pictures were thus taken of the skin in a relaxed state without splints. ImageJ [34] was used to delineate the outer contours of the wound. These values were expressed as a percentage of wound area compared to day 0 (right after surgery).

### 4.7. SPECT/CT Imaging and Biodistribution

SPECT/CT imaging, [^111^In]In-DTPA-PIC labelling, and analysis methods were carried out as described before [10]. Briefly, indium was complexed to MES-dissolved DTPA-functionalised PIC in a fridge (4 °C) for over 3 h. Then, ethylenediamine-tetra-acetic acid (EDTA, Sigma Aldrich) was added to chelate any unlabelled ^111^In and the solution was diluted to 4 mg/mL using PBS. Applied doses were 7.8 ± 1.3 MBq on day 0, 6.0 ± 0.3 MBq on day 1, and 4.3 ± 0.3 MBq on day 2. When analysing the data, the SPECT signal originating from the wounds was quantified using Inveon Research Workplace (Siemens Healthcare, The Hague, The Netherlands) by positioning regions of interest on the wounds in the CT scan. The amount of signal was compared before-and-after washing, after correction for natural decay, and expressed as the amount of signal remaining. As each mouse bore two wounds, two wounds were analysed. Biodistribution analysis after dissection was also performed, as described previously [10].

### 4.8. Tissue Harvesting

After sacrifice, a large skin flap surrounding and containing the wounds was excised using scissors and a scalpel. In the imaging study, wounds and control skin were harvested from this flap using a Ø 6 mm biopsy punch and then placed in 70% ethanol. Biodistribution analysis was first performed, and then the ^111^In was allowed to decay to safe values in 4% formaldehyde before processing the wounds for histology. In the main healing study, right wounds were harvested similarly and fixated immediately in 4% formaldehyde.

### 4.9. (Immuno-) Histology

Harvested tissue was fixated in 4% formaldehyde at room temperature for 24 h and then dehydrated and embedded in paraffin. Staining protocols for HE, Gr-1, F4/80, αSMA, and AZAN were identical to those published previously [9,10]. Von Kossa stainings were performed as follows: histological sections were deparaffinised in a xylene-alcohol-milliQ series, exposed to 5% silver nitrate under UV-illumination for 30 min, rinsed with milliQ, fixed with 2% sodium thiosulfate, and ultimately counter-stained with nuclear fast red. AZAN-stained images were scanned using a Pannoramic P250 digital slide scanner (3DHISTECH Ltd., Budapest, Hungary). Using Pannoramic Viewer (v1.15.4, 3DHISTECH Ltd.) software, one representative section was selected per sample, cropped, and exported at the highest quality settings for further analysis. All other histological sections were analysed under a microscope (Leica DM LB, Leica Microsystems B.V., Amsterdam, The Netherlands). Representative sections were photographed at 100×, 200×, or 400× zoom levels and (optionally) stitched together using a Zeiss Imager Z1 and an AxioCam MRc5 camera (Carl Zeiss Microimaging GmbH, Göttingen, Germany) using AxioVision V4.8.2. software’s MosaiQ plugin.

Histological results were scored blindly by authors RCV and FADTGW and presented as an average of the two. For all scorings, four representative sections of varying depth from each sample were investigated. Epithelial migration was scored on a scale of 0 to 4 in HE sections (no migration (0), partial migration (1), complete migration with no/partial keratinisation (2), complete migration with complete keratinisation (3), and hypertrophic (4)). The presence of PIC gel remnants in HE-stained sections was scored on a scale of 0 to 3 (absent (0), minor remnants (1), large remnants topically (2), and large remnants encapsulated within the wound repair tissue (3)). Cellular infiltration by granulocytes (GR-1 antibody), macrophages (F4/80 antibody), and myofibroblasts (αSMA antibody) were scored on a scale of 0 to 5 (cell type absent (0), mild presence (1), moderate presence (2), marked presence (3), very high presence (4), and wound tissue consists almost exclusively of this cell type (5)). Scores were treated as a scale, and a score of 0.5 could be awarded when the evaluator assessed the score to be slightly higher or lower than other sections of a similar score. The presence of giant cells in the HE sections was evaluated by eye. The percentage of blue-stained area in AZAN-stained sections, corresponding to collagenous structures, was calculated using ImageJ similarly as previously published [9]. However, instead of using colour thresholds to isolate the blue signal, the colour deconvolution plugin was used. Five sections were randomly selected to determine the vectors that could separate the different coloured tissues in the whole data set. This resulted in three images depicting only the blue (collagen), red (nuclei, erythrocytes, muscle, glial tissue), or green/grey (artefacts) coloured pixels. The analysis was performed as follows: area deemed as wound tissue was isolated; healthy tissue parts and volumes of PIC gels were then cropped out; and, ultimately, the blue-positive area in the total wound tissue deemed as ‘Collagen content’ was calculated and expressed as a percentage.

### 4.10. Statistical Analysis

Statistical analysis was performed using IBM SPSS software (version 20, Armonk, NY, USA). In the imaging study, the results of histological investigations were compared between left and right wounds using the paired student’s t-test. In the main wound healing study, the control group was compared to the single PIC and to the refreshed PIC group. In addition, the single PIC and refreshed PIC groups were compared. Data were split by time point during analysis, as only the inter-group differences were of interest. Statistical tests were selected based on the nature and behaviour of the acquired data. The rate of wound closure and the histological scorings of granulocytes, macrophages, and collagen content were analysed by one-way ANOVA with Tukey post hoc test. The migration of epithelium and histological population of myofibroblasts were tested with the non-parametric Kruskal–Wallis test. The presence of PIC gel in HE-stained sections was analysed using Cross Tabulation and Fisher’s Exact Test. The statistical significance level α was set at 0.05.

## 5. Conclusions

The present work has demonstrated the potential of PIC hydrogel as wound dressing material for treating splinted murine full-thickness skin wounds up to 14 days of healing. It was observed that a dressing removal protocol consisting of a cold saline irrigation procedure could remove PIC gel from wounds. However, the efficiency is heavily dependent on the used technique and is further hampered by the wound model’s severe stringency. Applying the PIC hydrogel does not cause damage to the wound nor interrupt the wound repair process. Moreover, it was found that regularly removing and replacing the gel helps prevent or reduce the chance of the PIC gel becoming encapsulated within the wound tissue. Wounds where PIC was regularly refreshed performed similarly to control wounds and were even slightly smaller on day 14. Finally, some evidence was found that PIC polymers may trigger heterotopic calcifications between two to four weeks after application if the material remains in the wound bed. It was concluded that PIC wound dressings perform on par with current clinically used products, have several practical benefits, and allow for further therapeutic and functional optimisation. The versatile PIC gel thus likely benefits both clinicians and wounded patients in the future.

## Figures and Tables

**Table 1:** Overview of the different groups used for the experiments (see also Section 4.5).

	Control Group Sham Treatment; No Gel, Only Tegaderm and Bandage	Single PIC Group Single Dose of PIC after Surgery, with Tegaderm and Bandage	Refreshed PIC Group PIC Gel with Tegaderm and Bandage. PIC Is Washed Away and Reapplied Every 3rd and 4th Day (e.g., Days 3, 7, 10, 14, …)	[^111^In] In-DTPA-PIC Group PIC Gel with ^111^In label Applied Initially with Tegaderm and Bandage. Washed Away and Reapplied on Days 1 and 2, and Washed Away on Day 3	End-Points (Days after Surgery)
Part 1—Initial Study	*n* = 0	*n* = 3	*n* = 3	*n* = 0	14, 21, 28
Part 2—Washing and Imaging Study	*n* = 0	*n* = 0	*n* = 0	*n* = 5	3
Part 3—Main Study	*n* = 18	*n* = 18	*n* = 18	*n* = 0	3, 7, 14

## Data Availability

The raw/processed data required to reproduce these findings cannot be shared at this time due to technical or time limitations.

## Supplementary Materials

The following supporting information can be downloaded at: https://www.mdpi.com/article/10.3390/ijms24065127/s1.
