# Supplementary material for: Repeated Application and Removal of Polyisocyanopeptide Hydrogel Wound Dressings in a Splinted Full-Thickness Wound Model
Source: Int J Mol Sci. 2023 Mar 7;24(6):5127. doi: 10.3390/ijms24065127 (PMC10049480; doi:10.3390/ijms24065127)
Supplement: Supplementary file 1 [file ijms-24-05127-s001.zip › ijms-2209802-supplementary.pdf]

# Supplementary information:

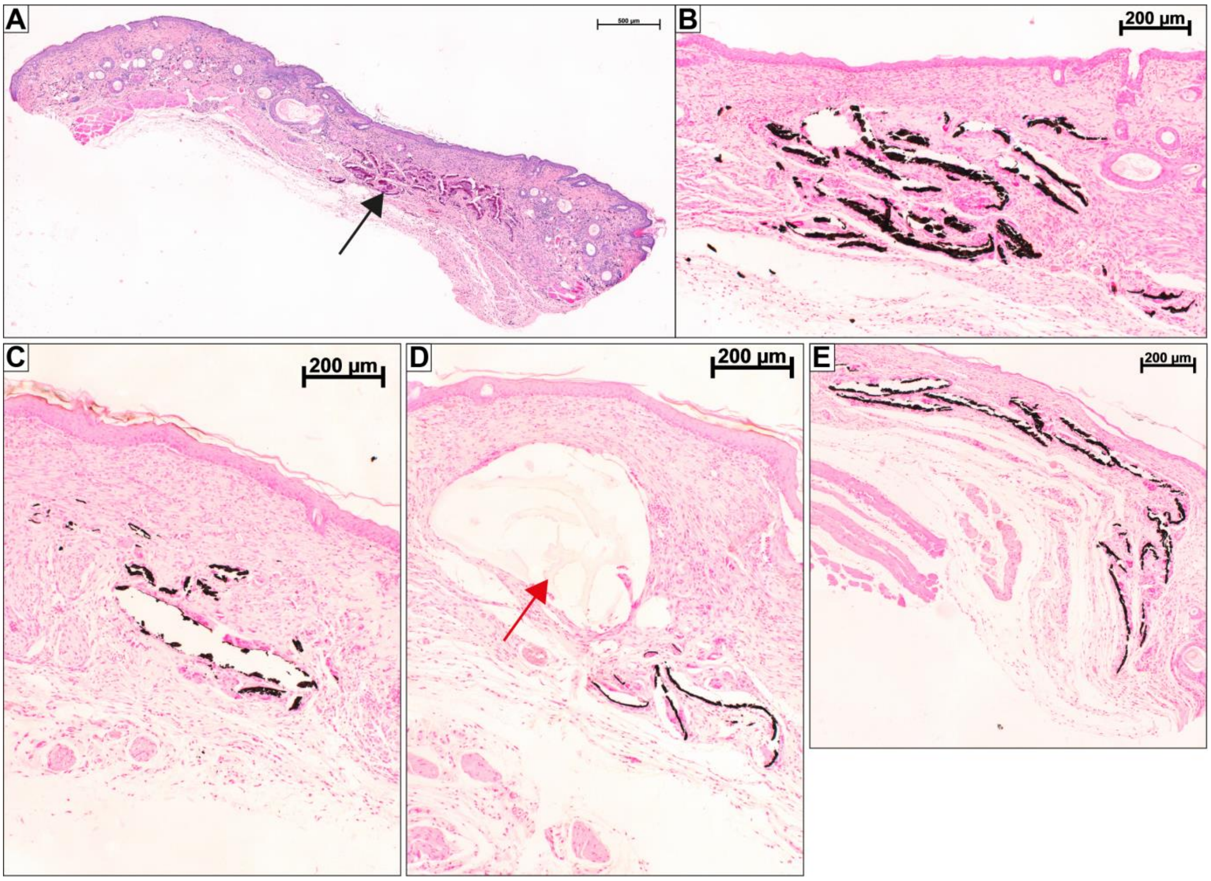


**Supplementary Figure S1:** Histological evaluation of the initial study. (A) Dense deposits, indicating a strong inflammatory response (black arrow), were identified on HE-stained sections (depicted is 28 days wounds subjected to the washing protocol). (B) Von Kossa stainings revealed these structures were calcified deposits (coloured black). (C) Calcified deposits were identified as early as 14 days after surgery. (D) This day 21 (no washing) sample displays both PIC gel (red arrow) and calcified polymer structures. (E) Calcified deposits on day 28 were also present in non-washed samples. Scale bars represent 500 µm (A) and 200 µm (B, C, D, E).

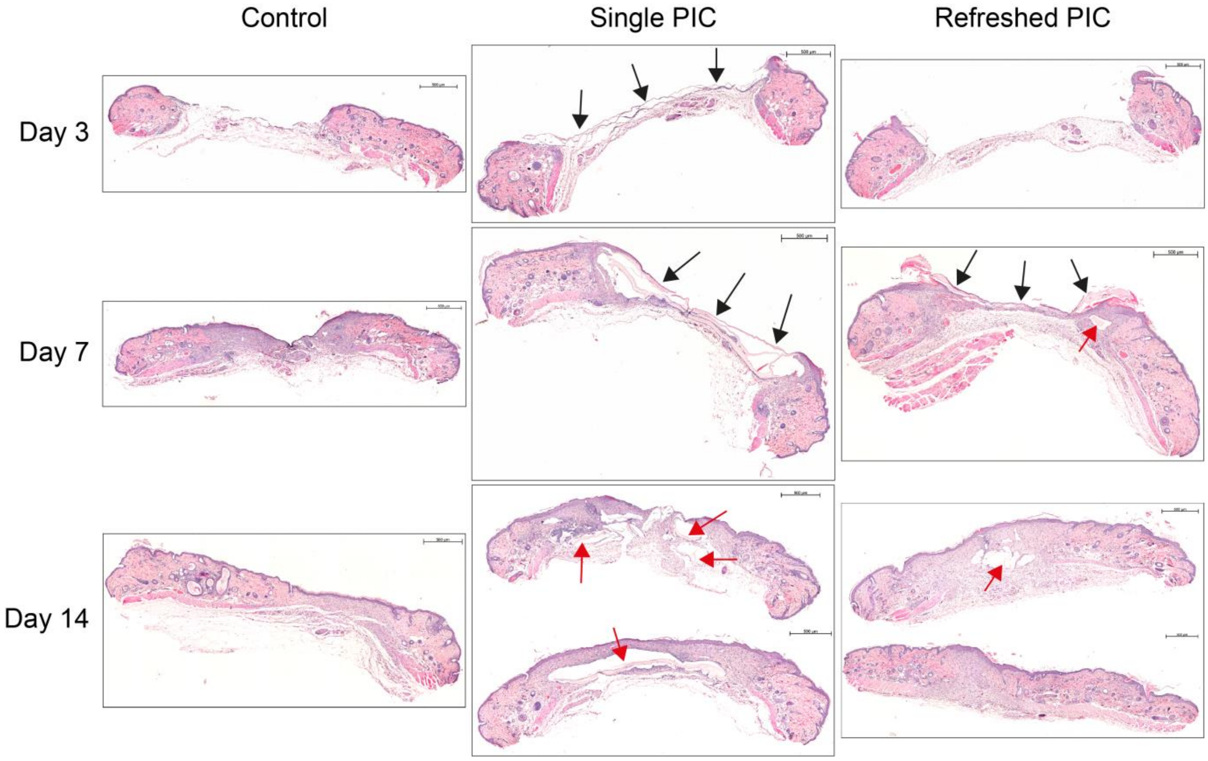


**Supplementary Figure S2:** An overview of representative HE-stained sections from each group and time point in the main wound healing study. On day 3, wounds tend to be large with minimal repair tissue formation. On days 7 and 14 the wounds close and the wound is repaired. Samples treated with PIC gel often, but not always, display PIC gel. This gel can be positioned topically (indicated with black arrows) or in compartments within the wound repair tissue (red arrows). Scale bars indicate 500 µm.
